# Fractional excretion of IgG in idiopathic membranous nephropathy with nephrotic syndrome: a predictive marker of risk and drug responsiveness

**DOI:** 10.1186/1471-2369-15-74

**Published:** 2014-05-08

**Authors:** Claudio Bazzi, Virginia Rizza, Daniela Casellato, Rafid Tofik, Anna-Lena Berg, Maurizio Gallieni, Giuseppe D’Amico, Omran Bakoush

**Affiliations:** 1 D’Amico Foundation for Renal Diseases Research, Milan, Italy; 2 Biochemical Laboratoryt, Azienda Ospedaliera Ospedale San Carlo Borromeo, Milan, Italy; 3 Department of Nephrology, Lund University, Lund, Sweden; 4 Nephrology and Dialysis Unit, Azienda Ospedaliera Ospedale San Carlo Borromeo, Milan, Italy; 5 Department of Internal Medicine, UAE University, Al Ain, United Arab Emirates

**Keywords:** Albuminuria, Idiopathic membranous glomerulonephritis, Immunoglobulin G, Steroids, Cyclophosphamide, ESRD, Nephrotic syndrome, Proteinuria, Treatment outcome

## Abstract

**Background:**

Treatment of idiopathic membranous nephropathy with nephrotic syndrome is still controversial. There is currently little known about the clinical use of renal biomarkers which may explain contradictory results obtained from clinical trials. In order to assess whether IgG-uria can predict the outcome in membranous nephropathy, we examined the value of baseline EF-IgG in predicting remission and progression of nephrotic syndrome.

**Methods:**

In a prospective cohort of 84 (34 female) idiopathic membranous nephropathy patients with nephrotic syndrome we validated the ability of the clinically available urine biomarker, IgG, to predict the risk of kidney disease progression and the beneficial effect of immunosuppression with steroids and cyclophosphamide. The fractional excretion of IgG (FE-IgG) and α1-microglobulin (FE-α1m), urine albumin/creatinine ratio, and eGFR were measured at the time of kidney biopsy. Primary outcome was progression to end stage kidney failure or kidney function (eGFR) decline ≥ 50% of baseline. Patients were followed up for 7.2 ± 4.1 years (range 1–16.8).

**Results:**

High FE-IgG (≥0.02) predicted an increased risk of kidney failure (Hazard Ratio, (HR) 8.2, 95%CI 1.0–66.3, p = 0.048) and lower chance of remission (HR 0.18, 95%CI 0.09–0.38, p < 0.001). The ten-year cumulative risk of kidney failure was 51.7% for patients with high FE-IgG compared to only 6.2% for patients with low FE-IgG. During the study, only 24% of patients with high FE-IgG entered remission compared to 90% of patients with low FE-IgG. Combined treatment with steroids and cyclophosphamide decreased the progression rate (–40%) and increased the remission rate (+36%) only in patients with high FE-IgG.

**Conclusion:**

In idiopathic membranous nephropathy patients with nephrotic syndrome, FE-IgG could be useful for predicting kidney disease progression, remission, and response to treatment.

## Background

Idiopathic membranous nephropathy (IMN) is a major cause of nephrotic syndrome (NS) in adult patients [1]. NS is associated with an increased risk of kidney failure and death from cardiovascular causes [2]. IMN is characterised by subepithelial localisation of IgG immune-complexes and lack of significant proliferation in the glomerular tuft. The recent discovery of the podocyte antigen PLA_2_R and its corresponding IgG autoantibodies confirmed the autoimmune nature of the disease [3].

There is no specific treatment for IMN. Early initiation of immunosuppressive treatment with steroids and cyclophosphamide has been recommended by some investigators [4,5]. Because of the high rate of spontaneous remission (about 30%), others suggested an initial conservative approach [6,7]. Immunosuppressive treatment could be harmful, but waiting until the condition starts to progress carries the risk of missing the “window of opportunity” for treatment [8-10]. Once this assessment is made, an individualised treatment based on risk *versus* benefit of the immunosuppressive treatment can be advocated [11].

Nowadays, the severity of kidney disease is commonly staged according to the kidney function estimated from serum creatinine (eGFR) [12]. However, eGFR is not an early predictive marker for treatment decisions [9]. Therefore, the search continues for early predictive markers that can estimate the risk of kidney disease progression. The ideal prognostic biomarker should be accurate and easy to implement in clinical practice. Such a biomarker could guide clinical decisions to avoid unnecessary treatment of patients who are not at risk of progression and to avoid unnecessary delay in initiation of treatment for those at high risk of progression to kidney failure.

As in other types of glomerular diseases, the primary main lesion in IMN is alteration of the glomerular filtration barrier (GFB) function with increased excretion of albumin (molecular radius, r =36 Å) and high molecular weight proteins such as IgG (r = 55 Å) and IgM (r = 120 Å) [13,14]. Thus, increased urine IgG concentrations in nephrotic patients could reflect activity and severity of the glomerulonephritis [13,15,16]. Recent studies have shown that IgG-uria could provide early prognostic information for patients with glomerular disease [13,15,17,18]. However, its value in predicting the remission and treatment outcome is still unclear. Using the data from the Milano and Lund glomerulonephritis longitudinal cohorts, we assessed whether IgG-uria can predict the functional outcome (remission *versus* progression) in patients with IMN and NS. We also evaluated the ability of IgG-uria to identify patients who might benefit from immunosuppressive treatment.

## Methods

### Patients

The cohort was derived from patients with IMN and NS diagnosed between January 1992 and December 2005 at the Nephrology Unit of San Carlo Borromeo Hospital, Milan, Italy (n = 70) and the Nephrology Department of Lund University, Sweden (n = 16). The inclusion criteria were nephrotic range proteinuria (24-hour proteinuria ≥ 3.5 g, or urinary albumin/creatinine ratio ≥ 2.0 g/g); serum albumin < 3.0 g/dL; baseline sCr <2.7 mg/dL and eGFR ≥24 ml/min/1.73 m^2^). The morphological diagnosis was in all cases established by light microscopy and immunofluorescence staining of representative kidney biopsy specimens containing at least six glomeruli. One histo-pathologist in each study center scored semi-quantitativly the tubulo-interstitial fibrosis as normal interstitium, focal or diffuse tubule-interstitial fibrosis, and the percentage of global glomerulosclerosis (GGS) was calculated. The patients did not have clinical or laboratory signs of secondary causes of IMN, such as systemic lupus erythematosus, connective tissue diseases, cancer, or medication with gold or penicillamine. The collection of 24-hour urine was done the day before the kidney biopsy, and the blood samples and the second voided urine specimens were obtained in the morning of the day of renal biopsy. The study complied with the Declaration of Helsinki and the local requirements for ethical approval (Lund regional ethical committee: LU 47-02). All patients gave informed written consent. The baseline characteristics of the cohort are in Table 1.

**Table 1:** Baseline characteristics of patients with idiopathic membranous nephropathy and nephrotic syndrome, classified according to fractional excretion of IgG

	**All patients**	**FE-IgG**		**P-value**
		**Low (<0.020)**	**High (≥0.020)**	
No. of patients	84	40	44	
Age (years)	55 ± 16	52 ± 15	58 ± 17	0.07
Sex (M/F)	50/34	20/19	30/15	0.18
eGFR (ml/min/1.73 m^2^)	72 ± 26	88 ± 20	58 ± 23	< 0.001
eGFR < 60 ml/min/1.73 m^2^	29 (34%)	5 (13%)	24 (53%)	<0.001
BP ≥140/90 mmHg	45 (54%)	13 (34%)	32 (71%)	0.001
Serum albumin g/L	23 ± 6	25 ± 5	22 ± 6	0.02
ACR mg/mmol	373 ± 256	243 ± 278	486 ± 177	< 0.001
FE IgG	0.052 ± 0.063	0.009 ± 0.005	0.090 ± 0.066	< 0.001
FE α1m	0.371 ± 0.409	0.124 ± 0.106	0.601 ± 0.447	< 0.001
GGS (%)	10 ± 14	6 ± 10	13 ± 17	0.02
TIF score	1.3 ± 1.3	0.7 ± 0.7	1.2 ± 0.7	0.08
Follow-up (months)	86 ± 50	98 ± 51	77 ± 48	0.055
ACE inhibitors treatment	50 (60%)	26 (65%)	24 (55%)	0.27

P-values are for the difference between the FE-IgG subgroups.

ACE: Angiotensin converting enzyme; eGFR: estimated GFR; BP: blood pressure; ACR: urinary albumin/creatinine ratio; FE IgG: fractional excretion of IgG; FE α1m: fractional excretion of α1-microglobulin; GGS: global glomerular sclerosis; TIF: tubulo-interstitial fibrosis.

### Treatment and follow-up

Thirty-five patients were treated only with supportive therapy, such as diuretics, antihypertensives, angiotensin enzyme inhibitors (ACEi/ARBs), statins, anti-platelet agents, and vitamin D3. Thirty-seven patients, besides supportive therapy, were treated soon after diagnosis with steroids and cyclophosphamide (St + Cyc) for six months according to the Ponticelli protocol: methylprednisolone 0.5–1.0 g iv for 3 days at the beginning of months 1, 3 and 5, followed by oral prednisolone 0.5 mg/kg/day during months 1, 3 and 5, and cyclophosphamide 1–2 mg/kg/day during months 2, 4 and 6 (lower dose of both drugs for elderly patients and patients with low GFR) [4]. Twelve patients were treated with steroids alone: five with prednisone 1 mg/kg/day for 4–12 months, and seven with ACTH 1–2 mg weekly for 4–11 months. Patients were followed up until the last planned clinic visit in 2009. The primary outcome was progression to kidney failure defined as start of renal replacement therapy (ESRD) or reduction of eGFR by ≥ 50% of baseline. The secondary outcome was complete or partial remission of NS (proteinuria < 0.2 or < 2.0 g/day, respectively).

### Laboratory analysis

A blood sample and a second morning fresh urine sample were analysed for concentration of creatinine, albumin, IgG and alpha 1 microglobulin in the chemistry laboratory of Azienda Ospedaliera Ospedale San Carlo Borromeo, Milan, and in the chemistry laboratory of hospital of Lund. Serum creatinine (sCr) and urinary creatinine (uCr) were measured enzymatically and expressed in μmol/L. Serum and urinary IgG, albumin and α1-microglobulin (α1m) were measured by immunonephelometry method on a BNA nephelometer (Behring, Milan, Italy) using rabbit serum antihuman antibodies (Behring) [16], and by immunoturbidimetry using a Cobas Mira S system (Roche Inc.) [19].

### Calculations

Urinary albumin-to-creatinine ratio (mg/g) (ACR) is the ratio of urinary albumin (mg/L) to urinary creatinine (g/L); fractional excretion of IgG (FE-IgG) and α1m (FE-α1m), expressed per 100 ml of creatinine clearance, was calculated according to the formula FE-IgG = (urinary protein/serum protein) × (serum creatinine/urinary creatinine) × 100.

Glomerular filtration rate (eGFR) was estimated from serum creatinine using the Chronic Kidney Disease Epidemiology Collaboration (CKD-Epi) formula [12].

### Statistical analysis

All statistical analyses were performed using IBM SPSS software version 19.0. Data are expressed as means ± SD. The differences between the study groups were determined using the Mann-Whitney U test, or the unpaired t-test as appropriate. Proportions were compared by Chi-square analysis. Correlations were assessed with Spearman Rank test. Receiver Operating Characteristic (ROC) curve was used to determine the cut-off values for the predictors of kidney failure (Table 2). These cut-off values were applied to the patients to create two groups for each predictor - high risk and low risk. Patients were also divided into high and low eGFR groups using a cut-off of < 60 ml/min/1.73 m^2^ according to the K/DOQI guidelines. eGFR < 60 ml/min/1.73 m^2^ identifies patients with moderate to severe CKD. The sensitivity and specificity of these cut-off points were used to calculate the positive likelihood ratio (Table 2). Kidney survival analysis was performed using the Kaplan–Meier method. Survival time was calculated from the date of diagnosis. The time horizon for cumulative kidney survival rate was set at 10 years. Patients were censored at the time of death or at the end of follow-up. Patients with missing data were excluded (2 patients was missing alpha 1 microglobulin ). Log-rank test was used to assess the difference in survival. Univariate and multivariate Cox proportional hazards regression analysis was performed on the population as a whole and on the relevant treatment groups. The tested dependent variables were FE-IgG, FE-α1m, ACR and eGFR. Two-sided p <0.05 was considered statistically significant.

**Table 2:** Characteristics of receiver operating curves (ROC) for the investigated biomarkers: area under the curves (AUC), cut-off levels, sensitivity, specificity and likelihood ratio for predicting kidney failure (n 84) and No remission (n 70) in idiopathic membranous nephropathy patients with nephrotic syndrome

**Biomarkers**	**AUC**	**p-value**	**Cut-off**	**Sensitivity %**	**Specificity %**	**Likelihood ratio**
**Prediction of kidney failure**						
eGFR	0.27	0.003	60	32	25	0.42
FE IgG	0.77	< 0.001	0.020	95	59	2.3
ACR	0.75	< 0.001	4000	84	56	1.9
FE α1m	0.76	< 0.001	0.240	84	60	2.1
High FE IgG and FE α1m	0.784	<0.001	0.02 + 0.24	84	68	2.6
**Prediction of no remission**						
eGFR	0.26	0.001	60	41	11	0.46
FE IgG	0.82	< 0.001	0.020	91	72	3.3
ACR	0.73	< 0.001	4000	76	69	2.5
FE α1m	0.76	< 0.001	0.240	85	67	2.6
High FE IgG and FE α1m	0.83	<0.001	0.02 + 0.24	82	78	3.7

eGFR: estimated GFR (ml/min/1.73 m^2^); FE IgG: fractional excretion of IgG; ACR: urinary albumin/creatinine ratio; FE α1m: fractional excretion of α1-microglobulin.

## Results

The fractional excretion of IgG (FE-IgG) was measured in 84 (34 female) patients Table 1. The overall average follow-up time was 86 ± 50 months (range 12–202). During follow-up, 20 patients progressed to kidney failure, 14 patients to ESRD, and 6 patients to eGFR ≤50% of baseline.

FE-IgG was strongly related to the baseline eGFR (r: –0.654, p = 0.01) and last eGFR (r: –0.570, p = 0.01), and weakly with global glomerulosclerosis (r = 0.380, p = 0.001) and tubulo-interstitial fibrosis (r = 0.297, p = 0.01). The optimal cut-off level for high FE-IgG was ≥ 0.02, for high FE-α1m ≥ 0.24, and for high ACR ≥ 4000 mg/g uCr (Table 2). FE-IgG had a better likelihood ratio for prediction of kidney failure thanFE-α1m, ACR or eGFR (Table 2).

**Figure 1:**
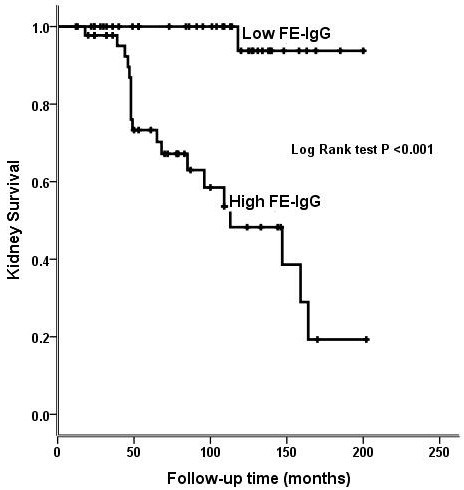
Kidney survival in patients with idiopathic membranous nephropathy and nephrotic syndrome according to FE-IgG levels.

The results of univariate Cox-regression analysis are shown in Table 3. In a multivariate Cox-regression analysis the association between FE-IgG and progression to kidney failure remains highly significant even after adjustment for the key potential confounding factors: age, kidney function, blood pressure, proteinuria, and treatment with ACEi/ARB and immunosuppression drugs, (HR 20.84, 95% CI: 2.8–156.8, p =0.003, Figure 1).

**Table 3:** Univariate Cox regression analysis for outcome of renal failure in 84 patients with idiopathic membranous nephropathy and nephrotic syndrome

**Variable**	**Beta**	**SE**	**P-value**	**HR**	**95% CI**
ACR (2 groups)	1.88	0.63	0.003	6.58	1.92–22.59
FE IgG (2groups)	3.08	1.03	0.003	21.76	2.90–163.11
FE α1m (2 groups)	2.14	0.64	0.001	8.53	2.46–29.62
eGFR (2 groups)	1.71	0.481	<0.001	5.5	2.14–14.13

Beta = regression coefficient, SE = standard error, HR = hazard ratio, CI = confidence interval, eGFR = estimated GFR < ≥ 60 ml/min/1.73, ACR < ≥ 4 g/g, FE-IgG < ≥ 0.020, FE-α1m < ≥ 0.24.

### Remission of nephrotic syndrome (NS)

**Figure 2:**
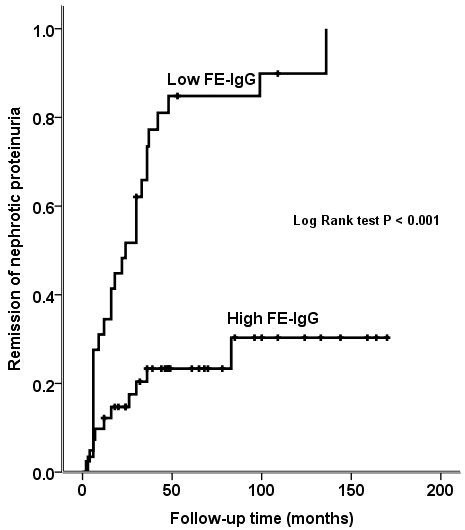
Remission of proteinuria in patients with idiopathic membranous nephropathy and nephrotic syndrome according to FE-IgG level.

Remission rate could be evaluated only for Milano group of patients (n = 70) (Figure 2). Irrespective of immunosuppressive treatment, most patients who had low FE-IgG went into remission (89.7% of patients at 36 months, 95% CI: 21–51). In contrast, the remission occurred only in less than a third of patients with high FE-IgG and at a later time (24.4% of patients at 128 months, 95% CI: 105–151, p <0.001).

#### FE-α1m groups

The remission was more frequent and earlier in patients with low FE-α1m (82.8% of patients at 47.1 months, 95%CI: 27–67 months) than patients with high FE-α1m (29.3%, at 119.9 months, 95% CI: 96–144, p <0.001).

#### ACR groups

The remission was more frequent and earlier in patients with low ACR (75.8% of pats, at 55.1 months, 95% CI: 33–77), than patients with high ACR (29.7%, at 109.5 months, 95% CI: 86–133, p < 0.001).

#### eGFR groups

Most of the patients with baseline eGFR ≥ 60 ml/min/1.73 m^2^ went into remission (69.6%, at 60.3 months, 95% CI: 41–79) compared to patients with baseline eGFR < 60 ml/min/1.73 m^2^ (16.7%, at 142.7 months, 95% CI: 118–167, p <0.001).

The multivariate Cox regression analysis identified only FE-IgG as an independent predictor for remission (HR 0.18, 95% CI, 0.09-0.38, p <0.001) (Figure 2).

### IgG fractional excretion and response to treatment

**Figure 3:**
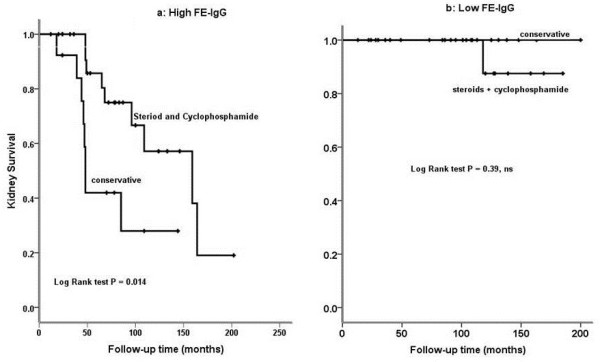
**Effect of immunosuppressive treatment on kidney survival in patients with idiopathic membranous nephropathy and nephrotic syndrome, according to FE-IgG level. a**: untreated patients with high FE-IgG were more likely than treated patients to progress to kidney failure. **b**: patients with low FE-IgG behaved similarly, progressing slowly to kidney failure.

The 37 patients treated with steroids and cyclophosphamide were compared with the 35 patients treated only with supportive therapy. The baseline characteristics of the two groups are in Table 4. There was no difference in initial kidney function or degree of proteinuria. During the study, the overall rates of progression to kidney failure in the untreated and treated patients did not differ significantly (37% *vs.* 27.6%, respectively, p = 0.84). However, untreated patients with high FE-IgG were more likely than treated patients to progress to kidney failure (72% *vs.* 43%, respectively, p =0.014) (Figure 3a). For patients with low FE-IgG there was no significant difference in risk of progression to kidney failure between the untreated and treated patients (12.5% and 0%, respectively, p = 0.39) (Figure 3b).

**Table 4:** Clinical, proteinuric and histologic parameters of 35 idiopathic membranous nephropathy patients with nephrotic syndrome not treated with immunosuppressive drugs and 37 others treated with combined steroids and cyclophosphamide

	**Untreated**	**Treated**	**p-value**
No. of patients	35	37	
Age (years)	53 ± 17	56 ± 16	0.65, ns
Sex (M/F)	16/19	26/11	0.034
Baseline eGFR ml/min/1.73 m^2^	77 ± 27	69 ± 25	0.19, ns
eGFR < 60 ml/min/1.73 m^2^	34%	32%	0.8, ns
BP ≥140/90 mmHg	52%	51%	0.89, ns
ACR mg/mmol	350 ± 242	379 ± 185	0.57, ns
FE IgG	0.040 ± 0.055	0.067 ± 0.069	0.07, ns
FE α1m	0.325 ± 0.382	0.435 ± 0.411	0.24, ns
GGS (%)	7.7 ± 10.7	11.3 ± 17.7	0.30, ns
TIF score	1.0 ± 0.9	1.4 ± 1.3	0.19, ns
Time to reach ESRD (months)	55 ± 29	90 ± 46	0.07, ns

Legend: eGFR: estimated GFR; ESRD: end stage renal disease; BP: blood pressure; ACR: urinary albumin/creatinine ratio; FE IgG: fractional excretion of IgG; FE α1m: fractional excretion of α1-microglobulin; GGS: global glomerular sclerosis; TIF: tubulo-interstitial fibrosis (0 absent, 1 focal, 2 diffuse), ns = non-significant.

Untreated and treated patient did not differ in overall remission rate (51.7% *vs.* 51.2%, p = 0.7). However, for patients with high FE-IgG, the remission rate was 0% for untreated and 36% for treated patients (p = 0.025). There was no difference in the frequency of remission between untreated and treated patients with low FE-IgG (93.7% *vs.* 84.6%, p = 0.23).

## Discussion

This longitudinal cohort study validated the predictive value of the fractional excretion of IgG in IMN patients with nephrotic syndrome. The heterogeneity of the progression to kidney failure in IMN patients and the lack of a reliable marker of disease severity has been a major confounding factor that contributed significantly to the contradictory results of the previous clinical treatment trials [20]. A recent meta-analysis of 1025 patients enrolled in 18 random controlled trials showed an increase in the likelihood of remission for patients treated with steroids and alkylating agents, but there were no beneficial effects on kidney function [21]. In our cohort, immunosuppressive treatment with steroids and cyclophosphamide significantly improved the clinical outcome only in patients with increased urinary excretion of IgG (FE-IgG ≥ 0.02). For these patients, immunosuppressive treatment reduced the 10-year incidence of kidney failure by 40% and increased the remission rate by 36%. Patients with lower concentrations of urine IgG (FE-IgG <0.020) went into spontaneous remission more frequently and maintained kidney function even without immunosuppressive treatment.

As in previous studies, our cohort showed that impairment of kidney function and increased proteinuria are associated with faster progression to kidney failure. However, the fractional excretion of IgG predicts both remission of NS (HR 0.18) and progression to kidney failure (HR 8.2) independently from albuminuria and GFR.

The role of albuminuria in the progression of kidney disease has been questioned in many experimental and clinical studies. The GFR estimated from serum creatinine is considered a late sign of the severity of kidney disease [22,23]. Thus, inclusion of FE-IgG in clinical practice guidelines may provide a substantial improvement in risk prediction during the early stages of IMN. It might help clinicians to individualise treatment and thereby improve outcome. Patients with high FE-IgG may benefit from more intensive monitoring of kidney function and from early initiation of immunosuppressive therapy.

The last few years have seen the development of various new immunosuppressive treatments, such as mycophenolate mophetil, tacrolimus, rituximab, and synthetic ACTH for patients not responding to steroids and alkylating agents [24-28]. It will be of clinical interest to include FE-IgG in future treatment trials.

The main strength of our study, the possibility of using a simple, practical renal biomarker that can be included in routine clinical care for kidney disease patients, is particularly relevant. Urine IgG measured in a spot urine sample and calculation of FE-IgG can be integrated into the routine laboratory investigation in most clinical laboratories. Nephrotic patients are usually managed by kidney specialist, and the results of this study can be generalised among different health care systems.

Calculating the risk of kidney failure in nephrotic patients is important for management decisions, and maintaining kidney function is usually associated with better survival. Whether immunosuppressive treatment improves patient survival should be addressed in multicentre longitudinal studies on large numbers of patients.

## Conclusions

In conclusion, in idiopathic membranous nephropathy patients with nephrotic syndrome, urinary excretion of IgG could be helpful, in addition to albuminuria, for assessment of the risk of disease progression and response to treatment. Validation of our results on a large external cohort and in new clinical trials is warranted.

## Competing interests

The authors declare that they have no competing interests.

## Authors’ contributions

CB and OB designed the study, analyzed the data, and draft the manuscript. VR, DC, RT, AB, MG, and GD contributed to the design of the study and drafting the manuscript. All authors read and approved the final manuscript.

## Pre-publication history

The pre-publication history for this paper can be accessed here:

http://www.biomedcentral.com/1471-2369/15/74/prepub
